# Crosstalk-Free Real-Time Precision Opto-Control of Biochemical Processes Through Intra-Pixel Optical Decoupling

**DOI:** 10.1002/cmtd.202500153

**Published:** 2026-02-28

**Authors:** Ishaan Kartik Singh, Bin Dong, Nikta Zafarjafarzadeh, Chi Zhang

**Affiliations:** 1 James Tarpo Jr. and Margaret Tarpo Department of Chemistry, Purdue University, West Lafayette, Indiana, USA; 2 Department of Physics and Astronomy, Purdue University, West Lafayette, Indiana, USA; 3 Purdue Institute for Cancer Research, West Lafayette, Indiana, USA; 4 Purdue Institute of Inflammation, Immunology, and Infectious Disease, West Lafayette, Indiana, USA

**Keywords:** crosstalk, fluorescence, reactive oxygen species, real-time precision optical control

## Abstract

The ability to regulate biochemical processes in live cells and whole organisms is critical when addressing fundamental biological questions. However, conventional chemical treatment methods overlook the spatial heterogeneity of these processes, offering only the ability to perturb a sample globally. Optical approaches, in contrast, can regulate biochemical activities with submicron spatial precision. Real-time precision opto-control (RPOC) is a recently developed technique that integrates laser scanning, chemical imaging, real-time decision making, and spatially precise optical regulation. RPOC uses chemically specific optical signals to trigger an action laser for selective regulation of dynamic chemical species. In RPOC, optical readout and treatment occur simultaneously, enabling continuous monitoring of chemical changes during perturbation. However, activation of the action laser can enhance fluorescent signals in the readout channel, creating a form of crosstalk that obscures the readout of chemical changes during optical treatment. To overcome this limitation, we introduce intra-pixel optical decoupling, a method that separates optical control and readout within each image pixel. This strategy preserves the simultaneous treatment-and-readout capabilities of RPOC while eliminating action-laser-induced crosstalk. As a result, chemical changes at the active pixels or surrounding areas can be accurately quantified during treatment, improving RPOC’s ability to probe local chemical changes over time.

## 1 | Introduction

Spatially precise control of intracellular chemical processes has long been a challenge due to the inherent heterogeneity of subcellular structures. Traditionally, such controls are achieved through treating cells with target-selective compounds. Although effective in many cases, this approach relies on the specificity of chemical interactions and is thus sensitive to off-target effects. Chemical treatment also lacks spatial selectivity, since freely diffusing molecules cannot be easily confined to specific subcellular regions.

As an alternative to chemical perturbation, optical treatment can be delivered to specific intracellular locations with high spatial precision. Chemical processes can then be regulated through diverse light–matter interaction mechanisms such as photoisomerization [1, 2], photoconformational switching [3, 4], photouncaging [5], photosensitization [6, 7], photobleaching [8, 9], and photoionization [10–12]. These mechanisms enable optical control over processes such as activating photoswitchable compounds [13–15], modulating light-sensitive ion channels [16, 17], releasing small molecules locally [18, 19], generating reactive oxygen species (ROS) [20, 21], and damaging subcellular structures [22, 23]. Spatial controls of these chemical processes provide a new understanding of site-specific biological functions and enable new ways of controlling biological responses.

Typically, these optical treatments can be delivered in point scanning or structured illumination approaches [24]. However, these conventional optical control methods generally require collecting an image first to guide the determination of the region of interest (ROI), followed by supervised and indiscriminative illumination within the ROI. As a result, their chemical specificity is limited, and they cannot precisely target highly dynamic molecular compartments or organelles. This leaves a critical gap in the automated selection and treatment of mobile molecular targets, as well as in understanding how local chemical processes evolve at the treatment sites during optical regulations.

To address these limitations, we have developed real-time precision optical control (RPOC), a technology that uses chemical-selective optical signals to activate opto-control solely at targets of interest during laser scanning [24–27]. RPOC is based on a closed-loop optoelectronic feedback system integrated into a laser-scanning microscope. In RPOC, signal detection, processing, decision-making, and laser activation all occur in real time within the same pixel [24]. The system identifies molecular targets and concurrently treats them in an unsupervised manner. This design eliminates the time gap between imaging and opto-control and allows precise treatment of highly dynamic organelles and chemical species. RPOC has been extensively integrated with confocal fluorescence microscopy, where fluorescence signals are used for both target selection and optical readout [26]. The laser responsible for optical regulation is referred to as the action laser, and pixels where the action laser is engaged are called active pixels (APXs). Recently, a supervised ROI selection method has been developed for RPOC, permitting diverse treatment methods for different ROIs and greatly enhancing experimental throughput [28]. Furthermore, pulsed femtosecond lasers have been employed as the action lasers to perform single-organelle microsurgery, induce localized ROS, or generate low-density plasma at specific intracellular compartments [29]. RPOC has been shown to be capable of locally switching the state of photochromic molecules [25], generating ROS [26], activating inhibitors to alter microtubule polymerization [26, 28], perturbing centrosomes to affect cell division [28], and enhancing protein dynamics analysis [28].

Despite these advances, a key challenge remains in fluorescence-based RPOC: action-laser-induced fluorescence enhancement. Accurate fluorescence readout is essential for studying laser-induced local chemical changes. However, because RPOC performs imaging and opto-control simultaneously, the action laser can excite the same fluorophores used for readout within APXs. This leads to enhanced fluorescence on those pixels, which creates crosstalk that obscures the signal containing information relevant to the effects of treatment and complicates quantitative analysis.

To address this issue, we introduce an intra-pixel optical decoupling strategy that temporally separates the action-laser activation window and the fluorescence readout window within each pixel. This approach eliminates action-laser-induced fluorescence enhancement while preserving both optical treatment and detection at the same pixel on a microsecond timescale. It is applicable in both the supervised, unsupervised, and the hybrid mode of RPOC for stationary or mobile molecular targets or organelles. We demonstrate this capability by applying RPOC to quantify photobleaching dynamics of fluorescent proteins and organelle dyes across different subcellular environments. This development provides a method to disentangle readout contributions from different laser sources and to accurately quantify the chemical changes induced by the action lasers during optical treatment.

## 2 | Results and Discussion

### 2.1 | Intra-Pixel Optical Decoupling for RPOC

**FIGURE 1 |:**
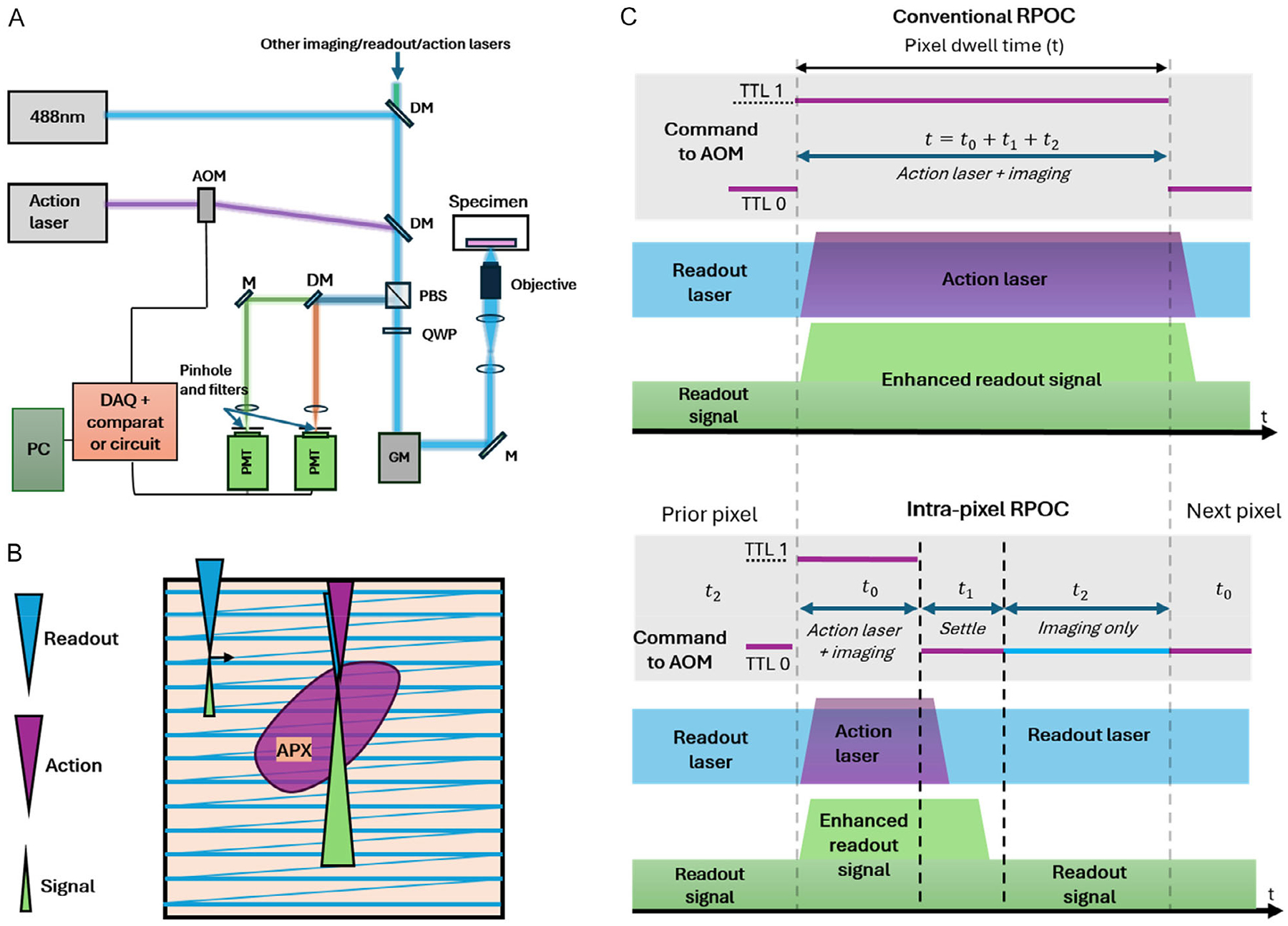
The working principle of intra-pixel optical decoupling for RPOC. (A) Optical schematic of the RPOC system. The setup is built on a custom inverted laser-scanning confocal fluorescence microscope. A 488 nm laser is used for excitation/readout, while the action laser is commanded by an AOM and combined into the optical path via a dichroic mirror (DM). Other imaging/readout or action lasers are also available. The AOM activation is synchronized with the imaging process through a multi-I/O DAQ unit controlled by custom Python code and by optical signals from the sample via the comparator circuit. A polarized beam splitter (PBS) and a quarter-wave plate (QWP) are used to separate fluorescence signals from the excitation beams. Signal detection is performed with photomultiplier tubes (PMTs), each combined with a pinhole and a bandpass filter. DM: dichroic mirror; GM: galvo mirrors; M: mirror; PC: computer. (B) Schematic of the raster scanning of excitation/readout lasers, signal generation/enhancement, and the action laser activation at the APXs. (C) Comparing the TTL commands, excitation/readout laser (blue), action laser (magenta), and fluorescence signals (green) in conventional RPOC and intra-pixel optical decoupling RPOC.

The operating principle of RPOC has been described in previous publications [24, 27]. Briefly, the system is based on a lab-built inverted confocal fluorescence microscope (Figure 1A). An excitation laser continuously raster scans the sample, and the detected optical signals are rapidly fed into a feedback loop involving a comparator circuit. When the desired signal criterion is met, the comparator circuit outputs a TTL high command to an acousto-optic modulator (AOM), which instantaneously activates the action laser within the same pixel, enabling optical regulation exclusively at this APX. When no desired signals are detected, the comparator circuit gives a TTL low command to the AOM, keeping the action laser off. Optical readout signals generated by either the excitation laser or dedicated readout lasers are recorded at all times to monitor sample responses.

When applying supervised ROI selection, the RPOC software can be used to delineate the area where action laser activation will occur (Figure 1B). A multi-channel I/O data acquisition (DAQ) interface synchronizes galvo scanning with AOM modulation of the action laser and ensures activation only when the scan is over selected ROIs. At APXs, the presence of the action laser may alter local optical signals, particularly enhancing fluorescence, which leads to significant crosstalk in the readout (Figure 1B,C). Such crosstalk has been observed in prior RPOC applications, such as mCherry-labeled proteins and MitoTracker-labeled mitochondria, leading to challenges in accurately quantifying chemical changes at APXs during treatment [28].

To address this issue, we developed an intra-pixel optical decoupling strategy that divides each pixel into three sequential segments (Figure 1C): (1) treatment + imaging; (2) settling; (3) imaging-only. The first segment, which occurs for a length *t*_0_ in each pixel, operates in the same manner as conventional RPOC. The multi-I/O outputs a TTL “1” signal to activate the AOM, and the pixel is optically treated with the action laser. After *t*_0_ elapses, the TTL command switches to “0”, leaving only the readout laser active. Limited detection bandwidth could cause lingering signal enhancement from the treatment phase, so for a time *t*_1_ after treatment, no signal is acquired. After that settling time, the optical signal is collected for a time *t*_2_ with only the readout laser activated. These 3 phases repeat over every pixel, making the overall dwell time the sum of *t*_0_, *t*_1_, and *t*_2_. The durations of *t*_0_ and *t*_2_ are time intervals for the treatment and pure imaging phases. They are selected based on the desired treatment length and readout signal-to-noise ratio requirement. The length of *t*_1_ is set by the temporal response of the optical and electronic system, and is pre-calibrated to be the shortest interval that fully suppresses residual signal enhancement before *t*_2_. Overall, the split of each pixel into these three phases enables optical readout to occur independent of optical treatment without sacrificing RPOC’s high-speed response speed and closed-loop feedback. The *t*_0_, *t*_1_, and *t*_2_ intervals are separately tunable in the system, dependent on the treatment or imaging requirements. It is generally applicable to all fluorephores that can be excited by the action laser. This acquisition scheme was implemented using custom Python-based DAQ and RPOC control software (Figure S1).

**FIGURE 2 |:**
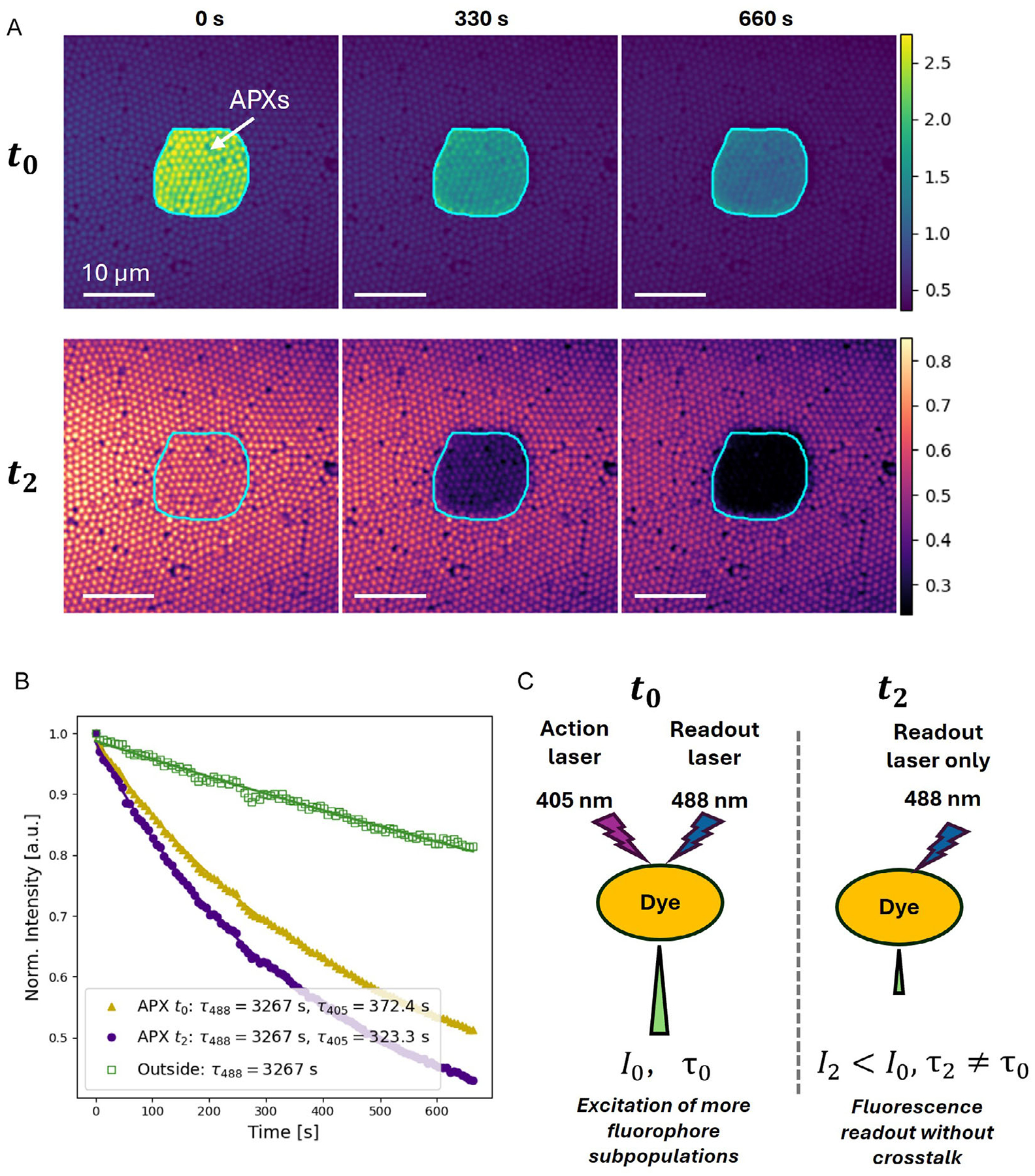
Eliminating action-laser-induced crosstalk during photobleaching of fluorescent beads. (A) Time-lapse images of fluorescent beads treated with a 405 nm laser. The top row shows images acquired in the *t*_0_ window, when the 488 nm laser is constantly scanning, and the 405 nm laser is only activated at APXs. The magenta outlined areas indicate the 405 nm treatment ROI and APXs. The bottom row shows images acquired in the *t*_2_ window, when only 488 nm is constantly scanning. (B) Normalized time-dependent fluorescence intensity curves from APXs in the *t*_0_, *t*_2_, and outside APXs in *t*_0_ in panel A. (C) A schematic illustrating the difference in intensity and signal decay time acquired in *t*_0_ and *t*_2_ time windows. I_0_, I_2_ are fluorescence signal intensity obtained in the *t*_0_ and *t*_2_ windows, and *τ*_0_ and *τ*_2_ are fluorescence signal decay time constants obtained from the *t*_0_ and *t*_2_ windows, respectively.

To evaluate the performance of the intra-pixel optical decoupling approach, we recorded the change in fluorescence signal from fluorescent beads (1 μm diameter, excitation: 488 nm, emission: 532 nm) due to photobleaching induced by a 405 nm action laser. The RPOC software was used to define an ROI for selective activation of the 405 nm laser (240 μW). The excitation/readout laser is 488 nm (20 μW). The pixel dwell time was set to 10 μs, with *t*_0_ = 3, *t*_1_ = 3, and *t*_2_ = 4 μs. When the 405 nm laser was activated simultaneously with the 488 nm imaging laser on APXs during the *t*_0_ period (Figure 2A, top row), the fluorescence signals of the beads were significantly enhanced. Such enhancement is absent for beads outside of APXs, and importantly, also absent for APXs during *t*_2_ (Figure 2A, bottom row). The raw fluorescence signal changes at APXs during *t*_0_ and *t*_2_, as well as exterior pixels, are shown in Figure S2. The 488 nm laser alone induced weak photobleaching on the exterior pixels, yielding a slow signal decay constant of 3267 s (Figure 2B). At the APXs, the presence of the 405 nm laser significantly accelerated the photobleaching. Since this process occurs in addition to photobleaching due to the 488 nm readout laser, the overall profile in APXs was bi-exponential, with the longer time constant matching the mono-exponential decay observed in the exterior pixels. When measured during *t*_0_, the fluorescent signal of APXs showed a secondary time constant of 372.4 s, whereas during *t*_2_, the constant was 323.3 s. This observed difference in fluorescence decay time constants between the *t*_0_ and *t*_2_ windows within the ROI likely arises from excitation of distinct fluorophore subpopulations by the 405 nm laser, which adds a slower component on top of the 488 nm readout. Since the chemical properties of those fluorophore subpopulations are not generally predictable, the photobleaching time constant extracted from the signal during *t*_2_ better quantifies the beads’ response over time to the 405 nm action laser than that from *t*_0_. These results demonstrate that the simultaneous presence of readout and action lasers could convolute quantification of fluorophore dynamics at APXs, and that intra-pixel optical decoupling effectively eliminates the crosstalk.

### 2.2 | Exploring the Photobleaching Dynamics of mCherry in Live Cells

Next, we applied intra-pixel optical decoupling in RPOC to photobleach mCherry in live cells. Here, mCherry was chosen as a case study because its action laser crosstalk was observed previously, and its photophysics are known to be complex and sensitive to oxygen availability. HeLa Cells were transfected with Histone-2-mCherry to visualize the nucleus via mCherry fluorescence. MCherry has an excitation maximum at 587 nm. However, excitation at this wavelength (and even at 532 nm) led to rapid photobleaching, even under low laser power. In contrast, 488 nm excitation produced minimal photobleaching. Consequently, a 488 nm laser (~20 μW at the sample) was used as the excitation and readout source for RPOC. The cells were prepared and placed in a stage-top incubator with an O_2_ level of about 20%.

**FIGURE 3 |:**
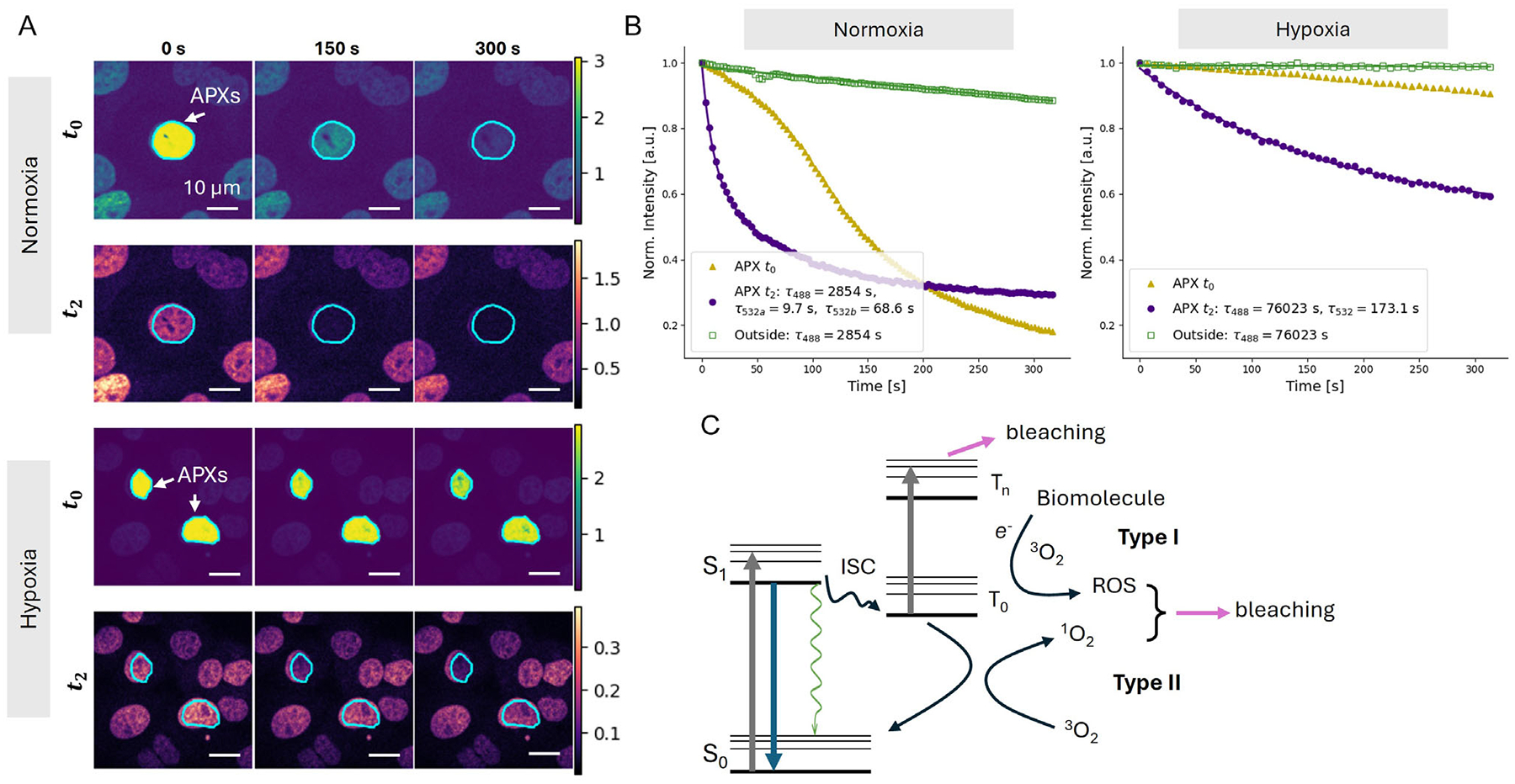
Extracting the photointeraction dynamics of the 532 nm laser with mCherry. (A) Time-lapse images of Histone2B-mCherry signals in HeLa cell nuclei under normoxia (20% oxygen) and hypoxia (0.1% oxygen) conditions during 532 nm laser treatment. The top and bottom rows in each case show images acquired in the *t*_0_ and *t*_2_ windows over time, respectively. The blue curves outline APX regions. (B) Normalized time-dependent fluorescence intensity curves extracted from the *t*_0_ and *t*_2_ windows for both treated and untreated nuclei regions in panel A. (C) Jablonski diagram illustrating photobleaching pathways, including triplet–triplet absorption and type I and type II photosensitization processes. ISC: intersystem crossing; ROS: reactive oxygen species; ^1^O_2_: singlet oxygen; ^3^O_2_: triplet oxygen.

To assess the photobleaching dynamics, a 532 nm laser (100 μW) was used as the action laser, and treatment regions were defined using the RPOC software (Figure 3A). Although strong fluorescence enhancement was observed during the *t*_0_ window upon 532 nm illumination, the *t*_2_ images showed no such crosstalk (Figure 3A, Video S1). Time-lapse fluorescence imaging from different regions and time windows revealed that excitation at 488 nm alone resulted in slow photobleaching, with a decay constant of about 2854 s (Figure 3B). Photobleaching induced by 532 nm excitation and probed with only 488 nm exhibited a rapid decay that was best fit with a tri-exponential function with time constants of 2854, 68.6, and 9.7 s. Notably, this decay could not be well fit using a bi-exponential model that includes the 2854 s time constant component. The inclusion of the 2854 s term accounts for the contribution from 488 nm excitation alone, while the two faster decay components likely reflect different photobleaching mechanisms under normoxia, such as type I and type II photosensitization, and triplet–triplet absorption (Figure 3C).

Interestingly, the signal extracted during *t*_0_ exhibited a sigmoidal shape not decomposable into a simple combination of exponentials. Two possible explanations for the unique photobleaching profile are (1) excitation of a subpopulation of mCherry molecules with distinct excitation-emission dynamics, and (2) Excitation of the mCherry at a relatively shorter wavelength. When excitation was shifted to 589 nm, redshifting the emission, the sigmoidal profile was no longer observed (Figure S3). However, the difficulty of precisely identifying the mechanism responsible makes it clear why intra-pixel decoupling of treatment from readout is necessary. By separating the fluorophore response from the action laser, direct quantitative analysis of local fluorophore kinetics during RPOC was made possible.

Strong photobleaching induced by the 532 nm laser likely involves local generation of ROS and singlet oxygen. To evaluate this possibility, we controlled the molecular oxygen level in the sample. Using the stage-top incubator, the O_2_ concentration was reduced to 0.1% during imaging and RPOC. Cells were first exposed to this extremely low-oxygen environment for 4 h to ensure effective depletion of dissolved oxygen, followed by RPOC experiments examining mCherry photobleaching under 532 nm illumination (Figure 3A). Strong crosstalk was also observed in the *t*_0_ time window, whereas no such crosstalk appeared in the *t*_2_ window (Figure 3A, Video S2). The resulting photobleaching time traces exhibited substantially longer decay constants under hypoxic conditions (Figure 3B), indicating that molecular oxygen contributes significantly to the bleaching process when present. The raw data curves are shown in Figure S4. This observation is consistent with the well-established mechanism of type I and type II photosensitization involving triplet-state conversion [30, 31]. In such processes, electron transfer involving molecular oxygen may produce ROS, and energy transfer from the triplet state of the fluorophore can generate singlet oxygen. These ROS and singlet oxygen would subsequently damage the fluorophore, leading to photobleaching (Figure 3C). It is worth noting that the ROS detected here are primarily laser-induced rather than biologically produced, since the fluorescence signal decay is action-laser-dependent. Besides these oxygen-associated photobleaching pathways, our results also suggest that the 532 nm laser can photobleach mCherry through O_2_-independent mechanisms. These processes likely include direct photochemical bond cleavage [32], triplet–triplet absorption [33, 34], and other nonradiative quenching processes. Controlling the oxygen level thus provides a way to disentangle molecular-oxygen-mediated bleaching from other photochemical pathways.

### 2.3 | Intra-Pixel Optical Decoupling RPOC for Mobile Mitochondria in Live Cells

**FIGURE 4 |:**
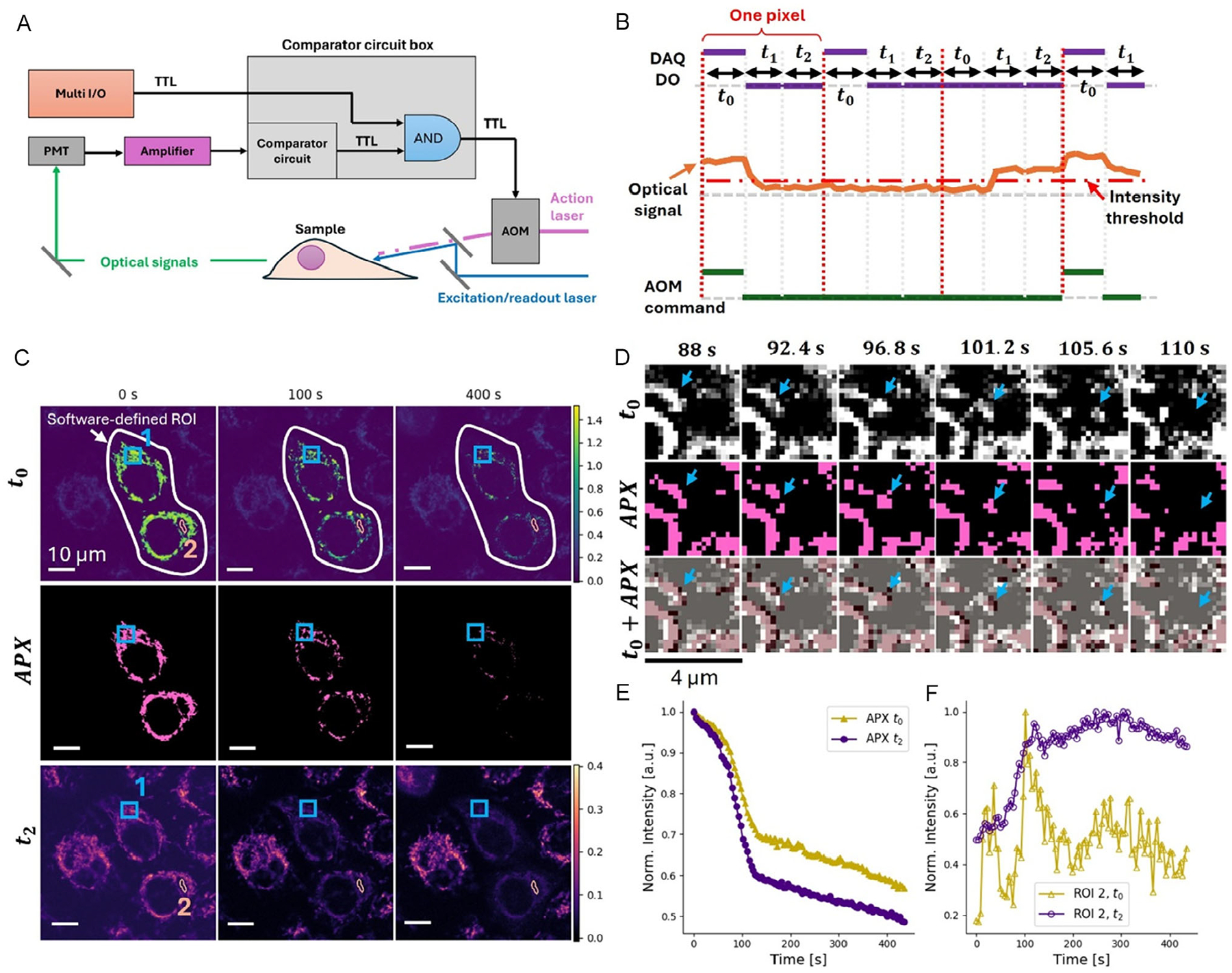
Intra-pixel optical decoupling for real-time treatment of mobile targets. (A) Schematic illustrating the hardware configuration and workflow combining the comparator circuit with the intra-pixel optical decoupling method. (B) Schematic showing the DAQ digital output (DO) command for intra-pixel decoupling, the optical signals relative to the intensity threshold, and the resulting AOM command after the digital logic AND operation. (C) Three time points of MitoTracker Red fluorescence images from MIA PaCa2 cells acquired during *t*_0_ and *t*_2_, as well as the corresponding APX associated with these time points. (D) Magnified regions at selected time points from panel C, ROI 1, highlighting the time-varying APXs associated with mobile mitochondria in selected cells. The blue arrows highlight mobile mitochondria receiving treatment as they move. Optical treatment pixels are diverse in each frame and automatically stop when the fluorescence signals drop below the intensity threshold. The overlay of *t*_0_ fluorescence signals and APX (light red) is shown in the bottom row. (E) Time-dependent fluorescence signal changes within APXs for *t*_0_ and *t*_2_. (F) Time-dependent fluorescence signal changes from panel C, ROI 2. The *t*_2_ curve signal increase reveals MitoTracker leakage from mitochondria into the cytosol over time, an effect not clearly revealed in the *t*_0_ curve from the same ROI due to the action laser crosstalk.

Intra-pixel optical decoupling can be easily integrated with a comparator circuit for real-time unsupervised optical treatment. This configuration is particularly suitable for precisely targeting dynamic molecular species or mobile organelles in live cells. The schematic of this process is shown in Figure 4A,B, with detailed circuit connections provided in Figure S5.

The TTL commands sent from the multi-I/O remain unchanged in this configuration and are generated from a preset mask, as in previous examples. However, these commands are no longer sent directly to the AOM to modulate the action laser. Instead, the detected optical intensity is compared with a predefined threshold to generate a secondary TTL signal. This signal is logically ANDed with the multi-I/O TTL command, and the resulting output controls the AOM. As a result, the action laser is only activated during *t*_0_, and only on pixels within the preselected ROI where molecular targets are detected (Figure 4B). This combined operation preserves the benefits of intra-pixel optical decoupling and further enables dynamic targeting and selective treatment of highly mobile targets within cells.

To demonstrate this capability, we used MitoTracker-labeled mitochondria as the mobile targets. MitoTracker Red CMXRos was known to have fluorescence signal enhancement by the 405 nm action laser. Mia PaCa2 cells were prepared, stained with MitoTracker, and cultured in a stage-top incubator with an oxygen level of about 20% for RPOC. The 488 nm laser served as both the excitation/readout laser for target selection and readout, while a strong 532 nm laser (~450 μW) was used as the action laser to perturb mitochondrial function. The time durations were set to be *t*_0_ = 3, *t*_1_ = 3, and *t*_2_ = 4 μs. Time-lapse fluorescence images acquired during t_0_ revealed significant MitoTracker signal enhancement induced by the 532 nm illumination (Figure 4C). Because mitochondria are highly dynamic, the APXs continuously change over time. Since the action laser was controlled by a logical AND with the real-time detected signal, the treated APXs align accurately with mitochondria even for rapidly moving ones (Figure 4D). When intra-pixel decoupling was applied, images from *t*_2_ showed no fluorescence crosstalk from the action laser, with accurate fluorescence signal changes visible over time (Figure 4C).

Continuous illumination of MitoTracker-labeled mitochondria resulted in disruption of the mitochondrial membrane potential and subsequent leakage of the dye into the cytosol, as evidenced by the time-dependent fluorescence changes observed at the APXs and in the surrounding cytosol regions. Intra-pixel optical decoupling effectively isolates fluorescence signals excited solely by the readout laser, enabling accurate monitoring of local photochemical responses during RPOC. By comparing fluorescence signal changes acquired on APXs in *t*_0_ and *t*_2_, we observed a sharp transition occurring at approximately 90–100 s in both traces (Figure 4E, Video S3). This sharp kink reflects a specific change in the mechanism contributing to fluorescent decay. This change was induced by the rapid leakage of MitoTracker into the cytosol, due to mitochondrial membrane damage. After this point, conventional photobleaching dominated the fluorescence decrease. This fast leakage was detected in both the *t*_0_ and *t*_2_ time windows (Figure 4E).

When selecting areas outside but adjacent to APXs (Area 2 in Figure 4C), it is possible to capture the rising fluorescence in the cytosol caused by dye leakage from mitochondria. However, fluorescence signals during *t*_0_ exhibit spikes and fluctuations due to transient increases in the MitoTracker signal that alter APX selection (Figure 4F). These crosstalk-induced signal fluctuations are eliminated during *t*_2_, providing smooth and crosstalk-free measurements of MitoTracker leakage into the cytosol (Figure 4F). The rapid signal increase around 90–100 s in the *t*_2_ signal (Figure 4F) results from the same MitoTracker leakage mechanism shown in Figure 4E. This result clearly shows the need for intra-pixel optical decoupling to clearly reveal molecular dynamics during treatment and to remove crosstalk.

## 3 | Discussion

The *t*_1_ window is included to fully extinguish the signal enhancement before pure readout during *t*_2_. There are various factors that might impact the optimal length of *t*_1_, primarily related to the response time of the detection system. For our setup, the PMT amplifier’s bandwidth has the greatest effect. When the amplifier bandwidth is set to ~300 kHz, *t*_1_ has to exceed 5 μs. With a bandwidth of 3 MHz, *t*_1_ could be reduced to below 2 μs. However, bandwidth increase comes at the cost of lower amplifier gain.

Although mCherry and MitoTracker were used in this study as representative examples, the intra-pixel optical decoupling method described here is generally applicable to any fluorophore that generates crosstalk under action-laser illumination during RPOC.

An alternative to intra-pixel optical decoupling is frame-by-frame optical decoupling, where the action laser is activated every other frame, leaving the intervening frames for readout only. However, this strategy reduces the temporal resolution of the fluorescence signal. More importantly, it is not ideal for “simultaneous” treating and monitoring the same mobile cellular compartments, such as mitochondria, because the targets in the treatment frames and the readout frames may not spatially correlate due to fast sample dynamics between frames. In contrast, intra-pixel optical decoupling performs both treatment and readout within microseconds, ensuring precise co-registration of the action-laser perturbation and the readout signal from the same mobile object.

## 4 | Conclusion

In this study, we developed an intra-pixel optical decoupling method that eliminates action-laser crosstalk in the readout channels of RPOC. By dividing each pixel dwell time into three sequential windows (an opto-control window, a settling window, and a readout-only window), the fluorescence enhancement induced by the action laser becomes fully isolated from the readout process. This enables simplified, cleaner signal acquisition and more accurate interpretation of action-laser-induced molecular changes, both at APXs and in regions outside the APX areas. The time-window splitting is implemented primarily through our custom DAQ and RPOC software, which integrates high-speed laser-activation and data-acquisition. Furthermore, this approach can be combined with comparator circuitry to eliminate action-laser crosstalk during real-time APX selection. We validated this methodology by using 405 nm or 532 nm action lasers to perturb fluorescence microparticles, mCherry-transfected histone-2 under normoxic and hypoxic conditions, and MitoTracker-labeled mitochondria. Together, these results demonstrate that intra-pixel optical decoupling is a powerful method for improving our understanding of how local molecular species and biological activities are perturbed by action lasers. It significantly enhances the performance of RPOC, enabling higher-quality probing of local chemical dynamics during optical regulation.

## 5 | Methods

### 5.1 | Sample Preparation

HeLa Kyoto EB3-EGFP cells and HeLa Kyoto EGFP-*α*-tubulin/Histone-2-mCherry cells were obtained from Biohippo. MIA PaCa2 cells were obtained from ATCC. Cells were cultured in Dulbecco’s Modified Eagle Medium (DMEM; ATCC) supplemented with 10% fetal bovine serum (FBS; ATCC) and 1% penicillin-streptomycin (Thermo Fisher Scientific). For imaging, cells were seeded in 35 mm glass-bottom dishes (MatTek Life Sciences) containing 2 mL of culture medium and incubated at 37°C in a humidified atmosphere of 5% CO_2_. When cells reached approximately 50–70% confluency, they were used for optical control and imaging experiments within a stage-top incubator. For mitochondria labeling, MitoTracker Red CMXRos (Thermo Fisher Scientific) was added to the culture medium at a final concentration of 200 nM. The cells were then incubated with the dye for 30 min at 37°C and 5% CO_2_, and then washed with warm culture medium once before use.

Fluorescence microparticles were purchased from Phosphorex Inc. (Lot No. 2051). The excitation and emission maximum wavelengths are 460 and 500 nm, respectively. The microparticles were deposited onto a glass coverslip and air-dried to form a layer of particles for photobleaching experiments.

### 5.2 | RPOC

RPOC was implemented on a custom-built confocal fluorescence microscope. Four continuous-wave lasers at 405, 488, 532, and 589 nm (CNI lasers) were spatially combined for signal excitation, RPOC, and fluorescence readout. The 405 and 532 nm lasers served as action lasers. Each action laser was equipped with an AOM (M1205-T80L-1 with 552F-2 driver, Isomet) to enable real-time laser activation control. The first-order beams from the AOMs were then combined collinearly with the excitation lasers.

A small fraction of each action laser was deflected by a glass coverslip and measured using photodiodes (PDA10A2, Thorlabs) to monitor APXs. The combined beams passed through a polarizing beam splitter (PBS251, Thorlabs) and a quarter-wave plate (QWP) (10RP44–1, Newport) before entering a high-speed 2D galvo scanner (Saturn-5, ScannerMax). Samples were scanned within a stage-top incubator (OTH-STXF-WSKMXCO2O2, Tokai Hit) mounted on a commercial microscope frame (IX73, Olympus). A motorized 3D translation stage (H117 with Motor Focus Drive and ProScan III, Prior) provided precise sample positioning. A water-dipping objective lens (UPlanSApo-S 60×, NA 1.20, Olympus) focused the laser beams onto the sample for both imaging and RPOC.

Fluorescence signals were collected using photomultiplier tubes (PMT, H7422–40, Hamamatsu) and amplified with a PMT4V3 amplifier (Advanced Research Instruments Corporation). Each PMT was paired with a 300 μm pinhole for confocal detection. Green fluorescence bead signals were collected using a 509 nm bandpass filter (FF01–509/22, Semrock), while mCherry and MitoTracker Red signals were detected using a 642 nm bandpass filter (ET642/80m, Chroma Technology Corporation). A multi-I/O device (PCIe-6363 with BNC-2120, National Instruments) synchronized laser scanning with RPOC operations. The system supports supervised mode via custom RPOC software, unsupervised mode via a comparator-based control box, and a hybrid mode integrating both. The RPOC software for local laser activation and DAQ was written in Python. Signals within each time window were averaged (in signal voltage) for quantitative analysis and image display. Fluorescence images, APXs, and time-dependent fluorescence changes were processed and visualized using Python scripts. Plots in the supporting information were generated using Origin 2019b.

## Supplementary Material

Supporting Information

Video S1

Video S2

Video S3

Additional supporting information can be found online in the Supporting Information section. **Supporting Fig. S1:** The Python-based GUI performs supervised ROI selection for RPOC and simultaneous image acquisition for readout. In the left panel, the *t*_0_ window shows strong fluorescence crosstalk from the action laser in the selected ROI, whereas this crosstalk is absent in the *t*_2_ window on the right. This GUI can be combined with the comparator circuit box for unsupervised opto-control of mobile targets detected in selected ROIs. **Supporting Fig. S2:** Raw fluorescence signals from beads outside the APXs, within the *t*_0_ window on the APXs, and within the *t*_2_ window on the APXs, corresponding to the images in Figure 2. **Supporting Fig. S3:** Normalized histone-2-mCherry signal changes during RPOC, using a 488 nm laser for excitation/readout and varying powers of a 589 nm action laser. **Supporting Fig. S4:** Raw intensity of mCherry fluorescence signal changes from treated and untreated ROIs in Figure 3. (A) In normoxia condition (20% O_2_). (B) In hypoxia condition (0.1% O_2_). **Supporting Fig. S5:** The connection configuration of the comparator circuit box that enables the combined use of the software-defined ROI and automated APX determinations within the ROI. Optical signals serve as ‘Analog signal input 1’, while the software TTL is delivered as ‘Software TTL’. **Supporting Video S1.** Time-lapse fluorescence signal changes of mCherry in HeLa cell nuclei in the normoxic condition, acquired in the *t*_2_ and *t*_0_ windows. One nucleus was selected for RPOC using a 532 nm action laser, while a 488 nm laser served as the excitation/readout source. **Supporting Video S2.** Time-lapse fluorescence signal changes of mCherry in HeLa cell nuclei in the hypoxic condition, acquired in the *t*_2_ and t_0_ windows. Two nuclei were selected for RPOC using a 532 nm action laser, while a 488 nm laser served as the excitation/readout source. **Supporting Video S3.** Time-lapse MitoTracker signals from MIA PaCa2 cells excited by 488 nm laser, acquired in the *t*_2_ and *t*_0_ windows. Two cells were selected for RPOC using 532 nm as the action laser. The APXs were selected by combining the software RPOC function and real-time APX determination using the comparator circuit box.

## Data Availability

The data and code that support the findings of this study are available from the corresponding author upon reasonable request.
