# Supplementary material for: Crosstalk-Free Real-Time Precision Opto-Control of Biochemical Processes Through Intra-Pixel Optical Decoupling
Source: Chem Methods. Author manuscript; Available in PMC 2026 Apr 2. (PMC13042463; doi:10.1002/cmtd.202500153)
Supplement: Supporting Information [file NIHMS2157902-supplement-Supporting_Information.pdf]

Supporting Information

Crosstalk-Free Real-Time Precision Opto-Control of Biochemical Processes through Intra-Pixel Optical Decoupling

#Ishaan Kartik Singh[1,2], #Bin Dong[1,3], Nikta Zafarjafarzadeh[1,3], *Chi Zhang[1,3,4]

[1]James Tarpo Jr. and Margaret Tarpo Department of Chemistry, Purdue University, 560 Oval Dr., West Lafayette, IN 47907, USA.
[2]Department of Physics and Astronomy, Purdue University, 525 Northwestern Ave., West Lafayette, IN 47907, USA.
[3]Purdue Institute for Cancer Research, 201 S. University St., West Lafayette, IN 47907, USA.
[4]Purdue Institute of Inflammation, Immunology, and Infectious Disease, 207 S. Martin Jischke Dr., West Lafayette, IN 47907, USA.

#These authors contribute equally to this work
*zhan2017@purdue.edu

Supporting figures

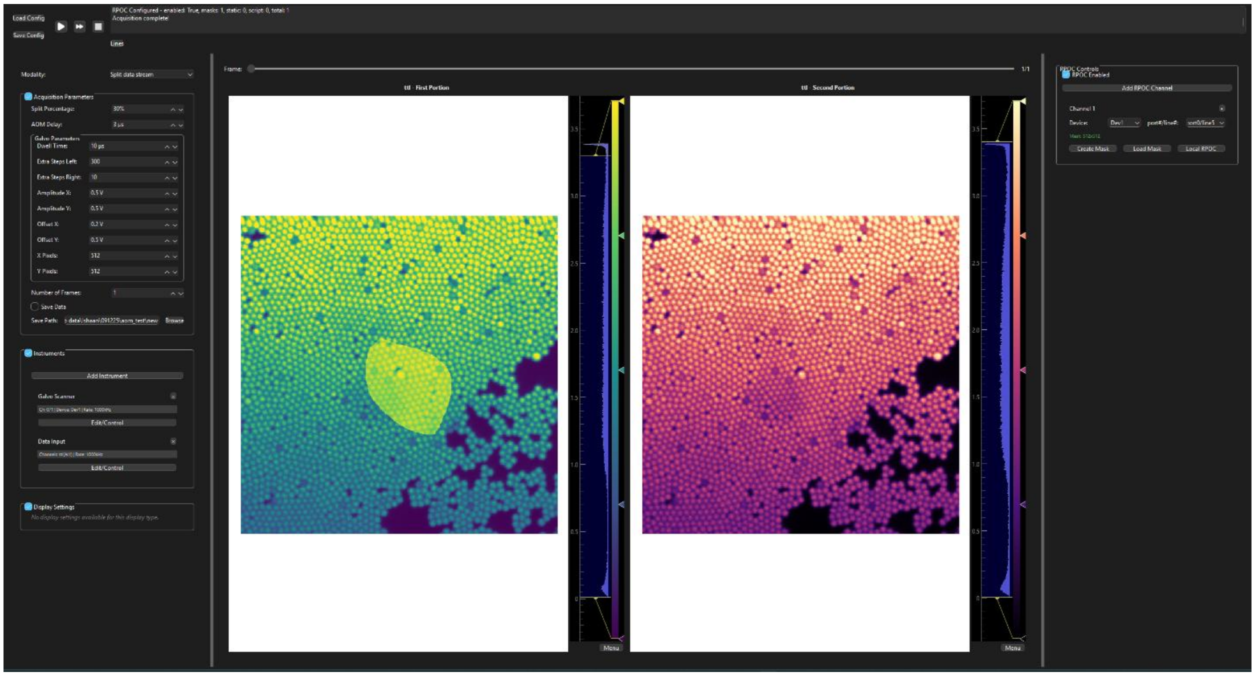

Figure S1. The Python-based GUI performs supervised ROI selection for RPOC and simultaneous image acquisition for readout. In the left panel, the t_0 window shows strong fluorescence crosstalk from the action laser, whereas this crosstalk is absent in the t_2 window on the right. This GUI can be combined with the comparator circuit box for unsupervised opto-control of mobile targets detected in selected ROIs.

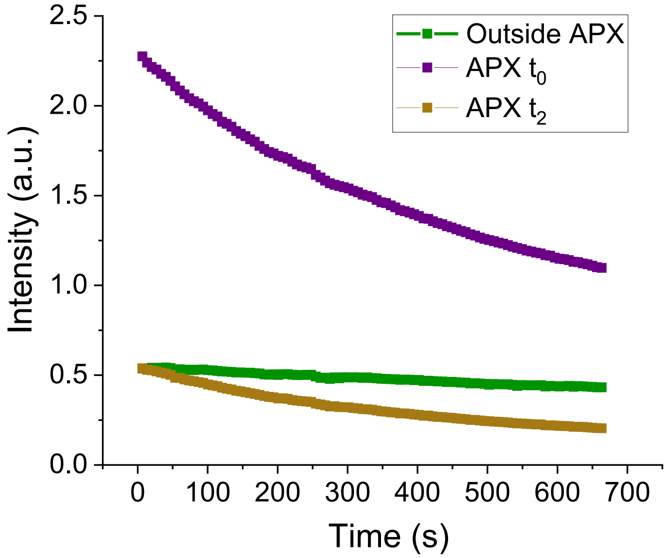

Figure S2. Raw fluorescence signals from beads outside the APXs, within the t_0 window on the APXs, and within the t_2 window on the APXs, corresponding to the images in Figure 2.

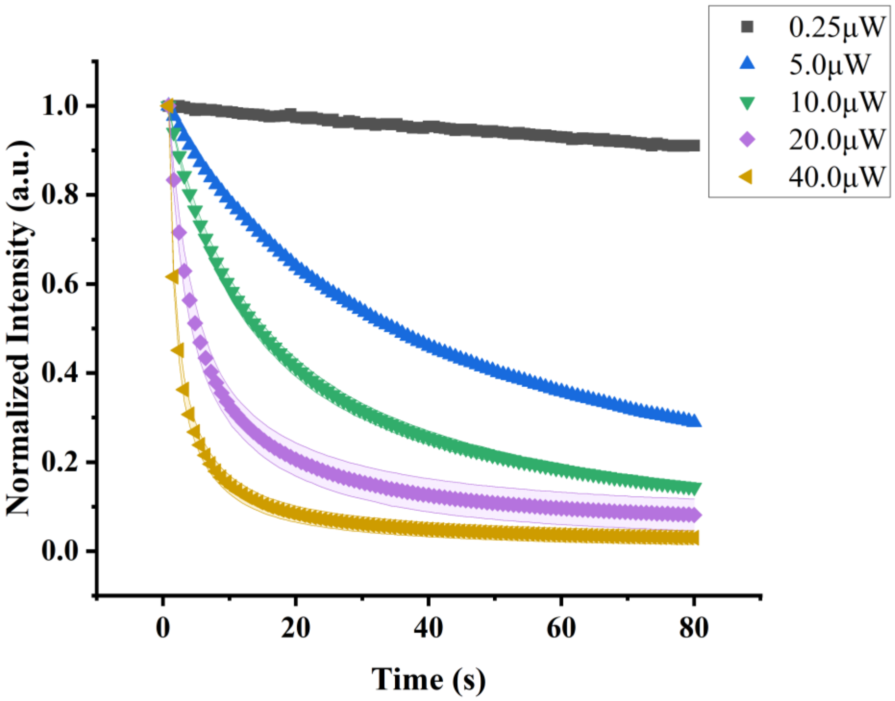

Figure S3. Normalized histone-2 mCherry fluorescence signal changes during RPOC using a 488 nm laser for excitation/readout and varying powers of a 589 nm action laser.

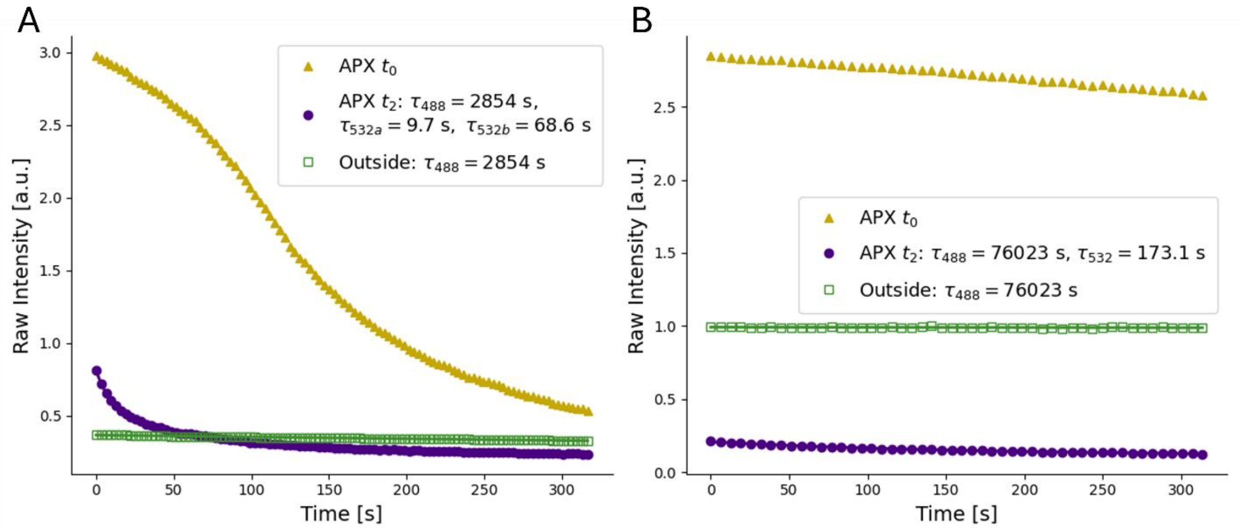

Figure S4. Raw intensity of mCherry fluorescence signal changes from treated and untreated ROIs in Figure 3. (A) In normoxia condition (20% O_2). (B) In hypoxia condition (0.1% O_2).

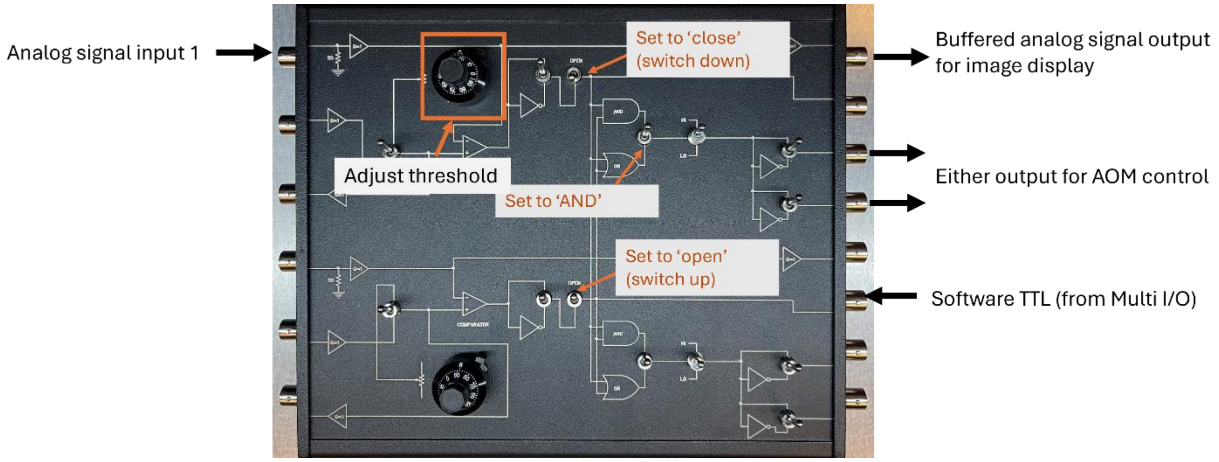

Figure S5. The connection configuration of the comparator circuit box that enables the hybrid use of the software-defined ROI and automated APX determinations within the ROIs. Optical signals serve as ‘Analog signal input 1’ port, while the software TTL is delivered as ‘Software TTL’.

Supporting Video 1. Time-lapse fluorescence signal changes of mCherry in HeLa cell nuclei in the normoxic condition, acquired in the t_2 and t_0 windows. One nucleus was selected for RPOC using a 532 nm action laser, while a 488 nm laser served as the excitation/readout source.

Supporting Video 2. Time-lapse fluorescence signal changes of mCherry in HeLa cell nuclei in the hypoxic condition, acquired in the t_2 and t_0 windows. One nucleus was selected for RPOC using a 532 nm action laser, while a 488 nm laser served as the excitation/readout source.

Supporting Video 3. Time-lapse MitoTracker signals from MIA PaCa2 cells excited by 488 nm laser, acquired in the t_2 and t_0 windows. Two cells were selected for RPOC with 532 nm action laser. The APXs are selected by combining the software RPOC function and real-time APX determination using the comparator circuit box.
